# Systemic and tumor-directed therapy for oligometastatic prostate cancer: study protocol for a phase II trial for veterans with de novo oligometastatic disease

**DOI:** 10.1186/s12885-019-5496-5

**Published:** 2019-04-01

**Authors:** Neil R. Parikh, Claudia Huiza, Jill S. Patel, Sonny Tsai, Nathisha Kalpage, May Thein, Sage Pitcher, Steve P. Lee, Warren S. Inouye, Mark L. Jordan, Homayoon Sanati, Lida Jafari, Carol J. Bennett, Greg E. Gin, Amar U. Kishan, Robert E. Reiter, Michael Lewis, Ahmad Sadeghi, William J. Aronson, Isla P. Garraway, Matthew B. Rettig, Nicholas G. Nickols

**Affiliations:** 1 Department of Radiation Oncology, UCLA, 200 UCLA Medical Plaza, Suite B265, Los Angeles, CA 90095-6951 USA; 2 VA Greater Los Angeles Healthcare System, Internal Medicine Service, Hematology/Oncology Section, 11301 Wilshire Blvd, Los Angeles, CA 90073 USA; 3 Department of Urology, UCLA, 27-139 Center for Health Sciences, Los Angeles, CA 90095 USA; 4 VA Long Beach Healthcare System, Radiation Oncology Service, 5901 East 7th Street, Long Beach, CA 90822 USA; 5 Department of Radiation Oncology, UCI, 101 The City Drive, Bldg. 23, Orange, CA 92868-3298 USA; 6 Department of Urology, UCI, 333 City Blvd W #2100, Orange, CA 92868 USA; 7 VA Long Beach Healthcare System, Urology Service, 5901 East 7th Street, Long Beach, CA 90822 USA; 8 VA Long Beach Healthcare System, Internal Medicine Service, Hematology/Oncology Section, 5901 East 7th Street, Long Beach, CA 90822 USA; 9 VA Greater Los Angeles Healthcare System, Imaging Service, Nuclear Medicine Section, 11301 Wilshire Blvd, Los Angeles, CA 90073 USA; 10 VA Greater Los Angeles Healthcare System, Urology Service, 11301 Wilshire Blvd, Los Angeles, CA 90073 USA; 11 VA Greater Los Angeles Healthcare System, Pathology Service, 11301 Wilshire Blvd, Los Angeles, CA 90073 USA; 12 VA Greater Los Angeles Healthcare System, Radiation Oncology Service, 11301 Wilshire Blvd, Los Angeles, CA 90073 USA

**Keywords:** Oligometastases, Prostate cancer, Stereotactic body radiotherapy, Abiraterone, Apalutamide, Leuprolide, Androgen deprivation therapy, Radical prostatectomy, Metastasis-directed therapy

## Abstract

**Background:**

The treatment paradigm for metastatic hormone-sensitive prostate cancer (mHSPC) patients is evolving. PET/CT now offers improved sensitivity and accuracy in staging. Recent randomized trial data supports escalated hormone therapy, local primary tumor therapy, and metastasis-directed therapy. The impact of combining such therapies into a multimodal approach is unknown. This Phase II single-arm clinical trial sponsored and funded by Veterans Affairs combines local, metastasis-directed, and systemic therapies to durably render patients free of detectable disease off active therapy.

**Methods:**

Patients with newly-diagnosed M1a/b prostate cancer (PSMA PET/CT staging is permitted) and 1–5 radiographically visible metastases (excluding pelvic lymph nodes) are undergoing local treatment with radical prostatectomy, limited duration systemic therapy for a total of six months (leuprolide, abiraterone acetate with prednisone, and apalutamide), metastasis-directed stereotactic body radiotherapy (SBRT), and post-operative fractionated radiotherapy if pT ≥ 3a, N1, or positive margins are present. The primary endpoint is the percent of patients achieving a serum PSA of < 0.05 ng/mL six months after recovery of serum testosterone ≥150 ng/dL. Secondary endpoints include time to biochemical progression, time to radiographic progression, time to initiation of alternative antineoplastic therapy, prostate cancer specific survival, health related quality-of-life, safety and tolerability.

**Discussion:**

To our knowledge, this is the first trial that tests a comprehensive systemic and tumor directed therapeutic strategy for patients with newly diagnosed oligometastatic prostate cancer. This trial, and others like it, represent the critical first step towards curative intent therapy for a patient population where palliation has been the norm.

**Trial registration:**

Clinicaltrials.gov identifier: NCT03298087 (registration date: September 29, 2017).

## Background

Metastatic prostate cancer is incurable, accounting for approximately 30,000 deaths in the United States each year [1]. Treatment intent is palliative. The core treatment is androgen deprivation therapy (ADT) consisting of GnRH analogs, which deactivate the androgen receptor (AR) through systemic lowering of androgen concentration. However, relapse on ADT is inevitable. Modern cohorts of patients treated with ADT alone have a median time to failure and overall survival of 11 and 42 months, respectively [2]. Patients are maintained on ADT continuously or intermittently until death.

New therapeutics that potently suppress AR signaling beyond conventional ADT include abiraterone acetate and apalutamide, which interfere with androgen synthesis and androgen receptor-androgen binding, respectively. These drugs are approved for castrate resistant prostate cancer [3, 4]. Two large randomized phase III trials also showed that addition of abiraterone acetate to ADT improved survival in patients with hormone sensitive metastatic prostate cancer (mHSPC), underscoring the role of escalated, early hormone therapy in the metastatic setting [5, 6].

Stereotactic body radiotherapy (SBRT) enables safe and accurate delivery of ionizing radiation to metastatic lesions with local control exceeding 95% for prostate cancer metastases [7]. A randomized trial showed that metastasis-directed therapy delayed time to initiation of ADT in patients with oligometastatic HSPC [8]. Additionally, recent data from the randomized Phase II SABR-COMET trial demonstrated improvement in survival in patients with a controlled primary malignancy and 1–5 metastatic lesions undergoing palliative intent standard of care with or without metastasis-directed therapy. Notably, 16 of 99 patients enrolled had prostate cancer [9].

The value of adding treatment to the primary tumor to ADT in mHSPC was suggested by retrospective analyses [10] and later by post-hoc subset analysis of oligometastatic patients from the randomized Phase III HORRAD trial [11]. This observation was then confirmed in the recent STAMPEDE trial that showed a survival advantage to addition of radiotherapy to the prostate to standard of care systemic therapy in a preplanned analysis of the enrolled oligometastatic patients [12].

A recent report of 20 patients with 5 or fewer metastases who underwent radical prostatectomy, ADT, and metastasis directed radiotherapy as part of a pilot program suggests a subset of patients may have a durable response to multimodal therapy [13].

The incidence of patients presenting at diagnosis with metastatic prostate cancer has risen [14]. A further increase in the frequency of patients diagnosed with de novo M1 prostate cancer is expected if and when PSMA PET probes receive FDA approval and are incorporated into routine care [15, 16]. PSMA PET/CT upstages a subset of patients to M1 who otherwise would be M0 by conventional imaging [17, 18].

With this confluence of treatment and diagnostic advances, the question arises whether a multimodal approach with aggressive, early treatment should be attempted with curative intent for oligometastatic prostate cancer patients. This single-arm trial, as well as others like it [10], tests the hypothesis that the combination of aggressive local, metastasis-directed, and hormonal therapy can achieve a durable undetectable disease burden in patients who are off therapy.

## Methods/design

### Trial design

**Fig. 1:**
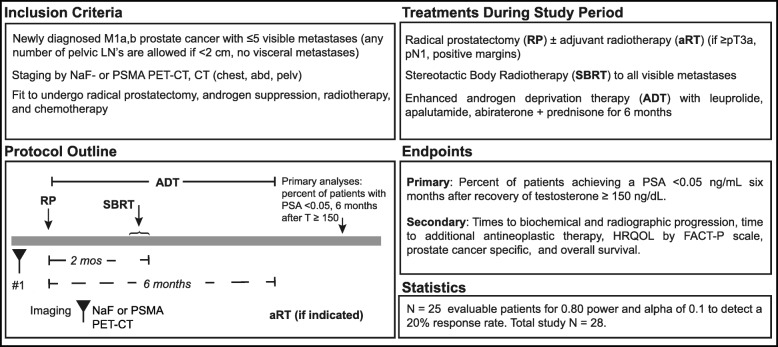
Trial Schema

The study is approved by the IRBs of VA Greater Los Angeles and VA Long Beach Healthcare Systems and is registered on clinicaltrials.gov (NCT03298087). The current protocol is version 3 dated March 26, 2018. The Department of Veterans Affairs (11,301 Wilshire Blvd., Los Angeles, CA 90073; 310–478-3711) is funding the study and Janssen is supplying apalutamide and abiraterone. Patients currently treated per standards of care will almost never achieve a durable undetectable PSA if systemic therapy is ceased and testosterone recovers. PSMA PET/CT may become FDA-approved within the next few years followed by adoption as part of routine staging. As such, we allow staging by PSMA PET/CT in this trial. Although not required, we anticipate that most enrolled patients will have undergone PSMA PET/CT. The trial schema is displayed in Fig. 1.

### Objectives

- Primary endpoint
  - Percent of patients achieving a serum PSA of < 0.05 ng/mL six months after recovery of serum testosterone ≥150 ng/dL
- Secondary endpoints
  - Time to biochemical progression
  - Time to radiographic progression (PCWG3 criteria)
  - Time to initiation of alternative antineoplastic therapy
  - Prostate cancer specific survival
  - Assessment of health related quality of life using the Functional Assessment of Cancer Therapy - Prostate (FACT-P) scale (http://www.facit.org/FACITOrg/Questionnaires)
  - Safety and tolerability (CTCAE v4)

### Inclusion criteria

- Biopsy confirmed diagnosis of prostate adenocarcinoma
- Age ≥ 18
- M1a/b disease with presence of 1–5 visible metastases (by NaF PET/CT or PSMA PET/CT, including CT of the chest, abdomen, and pelvis)
- Biopsy of one metastasis should be attempted, unless deemed not safe or feasible. If biopsy is not performed or is not diagnostic, then a second imaging modality must also be consistent with metastatic disease
- Patients may have any number of pelvic nodal metastases (but largest must be < 2 cm)
- Patient must be fit to undergo radical prostatectomy, to receive SBRT to all visible sites of metastases, and to receive ADT
- Total testosterone > 200 ng/dL prior to ADT
- Adequate performance status (ECOG 0–1)
- Clinical laboratory values at screening:
  - Hemoglobin ≥9.0 g/dL, independent of transfusion and/or growth factors within 3 months prior to randomization
  - Platelet count ≥100,000 × 10^9^/μL independent of transfusion and/or growth factors within 3 months prior to randomization
  - Serum albumin ≥3.0 g/dL
  - GFR ≥45 mL/min
  - Serum potassium ≥3.5 mmol/L
  - Serum total bilirubin ≤1.5 × ULN
  - Aspartate aminotransferase (AST) or alanine aminotransferase (ALT) < 2.5 × ULN

### Exclusion criteria

- Primary small cell carcinoma of the prostate
- Presence of visceral metastases
- Patients taking medications known to lower the seizure threshold (unless discontinued or substituted at least 4 weeks prior to study entry)
- Any evidence of spinal cord compression (radiological or clinical)
- Prior pelvic malignancy
- Prior pelvic radiation
- Concurrent malignancy aside from superficial skin cancers or superficial bladder tumors
- Inflammatory bowel disease or active collagen vascular disease
- History of seizure or known condition that may pre-dispose to seizure (e.g., prior stroke within 1 year to randomization, brain arteriovenous malformation, Schwannoma, meningioma, or other benign CNS or meningeal disease which may require treatment with surgery or radiation therapy)
- History of severe or unstable angina, myocardial infarction, symptomatic congestive heart failure, arterial or venous thromboembolic events (e.g., pulmonary embolism, cerebrovascular accident including transient ischemic attacks), or clinically significant ventricular arrhythmias within 6 months prior to randomization
- Current evidence of any of the following:
  - Uncontrolled hypertension
  - Gastrointestinal disorder affecting absorption
  - Active infection (e.g., human immunodeficiency virus [HIV] or viral hepatitis)
  - Baseline severe hepatic impairment (Child-Pugh Class B & C)
  - Any chronic medical condition requiring a higher dose of corticosteroid than 10 mg prednisone/prednisolone once daily
  - Any other condition that in the opinion of the investigator would preclude participation in this study
- If currently receiving treatment with the following drugs:
  - Treatment with CYP2D6 substrates that have a narrow therapeutic index. If an alternative treatment cannot be used, a dose reduction of the CYP2D6 substrate may be considered
  - Concomitant CYP2C8 inhibitors with narrow therapeutic index. If a concomitant CYP2C8 inhibitor with narrow therapeutic index must be co-administered, patients should be monitored closely for signs of toxicity related to the CYP2C8 inhibitor with a narrow therapeutic index if used concomitantly with abiraterone acetate
  - Concomitant strong CYP3A4 inducers. If a strong CYP3A4 inducer must be co-administered, abiraterone acetate dose frequency will be adjusted

### Selection and study enrollment procedures

Patients must be found to have metastatic prostate cancer without prior prostate cancer therapy. Patients are recruited from the urology, medical oncology, and radiation oncology clinics at the VA Greater Los Angeles and VA Long Beach Healthcare Systems, where the study has received IRB approval. Patients are consented by study investigators. Inclusion and exclusion criteria are determined by the study investigators. This is a single-arm study, with all patients enrolled to undergo treatment as below.

### Interventions

- Primary tumor therapies:
  - Radical prostatectomy, open or robot-assisted, with pelvic lymph node dissection. This surgery is performed by an experienced urologic surgeon and is the first treatment intervention, preceding the systemic and radiotherapies.
  - Post-operative pelvic RT. This is included if patients are found to have pT3a+, positive margins, or regionally involved nodes (N1). IMRT is delivered to the pelvic nodes (45–50.4 Gy) and prostate bed (66–72 Gy) in conventional fractionation according to RTOG contouring guidelines (modified as needed based on imaging). Gross disease may be boosted while respecting normal tissue dose constraints. This pelvic RT can extend beyond or be completed after ADT.
- Systemic hormonal therapies:
  - Patients initiate leuprolide, apalutamide and abiraterone acetate beginning immediately (within 0–3 days) after radical prostatectomy. Patients will receive a single dose of leuprolide, SQ, 45 mg, a six-month depot, and start apalutamide (ARN-509, 240 mg PO daily), and abiraterone acetate co-administered with prednisone (1000 mg PO and 10 mg PO daily, respectively), for a duration of six months.
- Metastasis-directed Stereotactic Body Radiotherapy (SBRT):
  - Patients undergo SBRT to all radiographically visible sites of M1a,b metastases within two months of initiation of ADT. A biologically equivalent dose (BED) to tumor of > 100 Gy (for an alpha-beta ratio of 3) is a goal but is not required [2] as constraints must be met [19]. Recommended SBRT dosing is displayed in Table 1.

**Table 1:** SBRT dose recommendations

Number of Fractions	Recommended Dose per Fraction
5 Fractions	6–8 Gy
3 Fractions	9–12 Gy
1 Fraction	16–18 Gy

### Follow-up and adherence to interventions

Scheduled treatment visits are every 30 days for the first year, and then every 3 months thereafter. Primary See Table 2 for schedule of evaluations. Each patient remains on study until unacceptable toxicity, disease progression, or withdrawal of consent, or two years. Even after completion of study, patients are offered routine post-trial care. Patients who leave the study prior to completion of therapy are not assessed for primary endpoint determination. Adherence to study drugs (apalutamide and abiraterone acetate) is monitored by pill count by study staff monthly during the six-month treatment period. Strategies to ensure adherence to study treatments and follow-up include ease of access to investigators and study staff by enrolled patients. Any clinical adverse event is recorded, managed as appropriate, and followed until resolution. Dose modifications for drug therapies are made based upon laboratory studies and observed toxicities. After completion of therapy, patients receive standard of care.

**Table 2:** Summary of study visits, procedures, and evaluations

	At inclusion	Day 1	Between day 30–60	At 6 months	Monthly for first 12 months	Every 3 months thereafter
NaF- or PSMA- PET CT (with CT of chest/abdomen/pelvis)	x					
PSA, total testosterone	x				x	x
Radical prostatectomy		x				
SBRT to all sites of metastases			x			
Prostate bed/nodes RT ^a^						
Lupron depot (6 month)^b^		x				
Start apalutamide, abiraterone acetate, prednisone ^b^		x				
Stop apalutamide, abiraterone acetate, taper prednisone				x		
Quality-of-life and toxicity assessments	x				x	x

^a^if indicated, This pelvic RT can extend beyond or be completed after ADT

^b^within 3 days of surgery

### Monitoring

The VA Clinical Sciences Research and Development (CSR&D) centralized Data Monitoring Committee (DMC) monitors this study. The DMC is a component of Veterans Affairs and independent of Janssen. The study investigators communicate directly with the DMC and supply the DMC progress reports every four months. Significant adverse events are reported promptly, including to the DMC, Janssen, VA IRB. The local VA IRB audits the trial annually. Plans for trial amendments are communicated to the IRB, DMC, sponsors. Amendments involving change of risk are additionally communicated to the subjects directly.

### Statistical analysis

#### Sample size

The current study hypothesis is that 20% of patients will achieve a PSA < 0.05 ng/mL six months after recovery of testosterone to > 150 ng/dL. A sample size of *N* = 25 evaluable patients has an 80% power to test the null hypothesis response rate of 5.5% against a two sided alternative response rate of 20% at a significance level (alpha) of 0.1. Assuming 10% of patients may not recover testosterone to ≥150 ng/dL, total study *N* = 28.

### Data analysis

The primary endpoint of our study, percent of patients achieving a serum PSA of < 0.05 ng/mL six months after recovery of serum testosterone ≥150 ng/dL, is based on PSA and testosterone labs that are checked every 30 days after completion of systemic therapy for six months, and then every three months thereafter. Survival times for secondary endpoints are defined from the day of enrollment until the event (e.g. biochemical progression, radiographic progression, initiation of additional antineoplastic therapy). Data is centrally coded and electronic data is stored in secure, password-protected computers at Veterans Affairs, behind locked doors. Paper data is stored in locked filing cabinets at Veterans Affairs, behind locked doors. The study investigators and staff have access to the final trial dataset. Trial results will be communicated through publications and presentations when analysis is complete.

## Discussion

The treatment paradigm for newly diagnosed oligometastatic HSPC is evolving rapidly. Recent data has shown the efficacy of intensified hormone therapy in addition to ADT [5, 6], treatment of the primary [12], and metastasis-directed therapy [8] – though each has as yet been studied in isolation. This study investigates the potential of a multimodal therapy combining each of these treatments. This effort is a first step towards curative intent therapy in a patient population that otherwise receives palliative intent therapy.

## Abbreviations

ADT: Androgen deprivation therapy
AE: Adverse event
ALT: Alanine aminotransferase
AR: Androgen receptor
aRT: Adjuvant radiotherapy
AST: Aspartate aminotransferase
BED: Biological effective dose
CBC: Complete blood count
CGH: Comparative genomic hybridizations
CNS: Central nervous system
CRPC: Castration resistant prostate cancer (mCRPC, m denotes metastatic)
CT: Computed-tomography
CTCAE: Common Terminology Criteria for Adverse Events
CYP: Cytochrome P450
ECOG: Eastern Cooperative Oncology Group
FACT-P: Functional Assessment of Cancer Therapy - Prostate
FDA: Food and Drug Administration
GFR: Glomerular filtration rate
GnRH: Gonadotropin-releasing hormone
HRQOL: Health-related quality of life
IMRT: Intensity-modulated radiation therapy
IRB: Institutional review board
mHSPC: Metastatic hormone-sensitive prostate cancer
MRI: Magnetic resonance imaging
NaF: Sodium Fluoride
NCI: National Cancer Institute
PET/CT: Positron emission tomography-computed tomography
PSA: Prostate specific antigen
PSMA: Prostate-specific membrane antigen
RNA: Ribonucleic acid
RP: Radical prostatectomy
RTOG: Radiation Therapy Oncology Group
SAE: Serious adverse event
SBRT: Stereotactic body radiotherapy
SQ: Subcutaneous
UCLA: University of California, Los Angeles
ULN: Upper limit of normal
VA: Veterans Affairs

## References

[1] Group., U.S.C.S.W. U.S (2018). Cancer Statistics Data Visualizations Tool, based on November 2017 submission data (1999-2015): U.S. Department of Health and Human Services, Centers for Disease Control and Prevention and National Cancer Institute.

[2] James ND (2015). Survival with newly diagnosed metastatic prostate Cancer in the "docetaxel era": data from 917 patients in the control arm of the STAMPEDE trial (MRC PR08, CRUK/06/019). Eur Urol.

[3] Smith MR (2018). Apalutamide treatment and metastasis-free survival in prostate Cancer. N Engl J Med.

[4] de Bono JS (2011). Abiraterone and increased survival in metastatic prostate cancer. N Engl J Med.

[5] Fizazi K (2017). Abiraterone plus prednisone in metastatic, castration-sensitive prostate Cancer. N Engl J Med.

[6] James ND (2017). Abiraterone for prostate Cancer not previously treated with hormone therapy. N Engl J Med.

[7] Ost P (2016). Progression-free survival following stereotactic body radiotherapy for Oligometastatic prostate Cancer treatment-naive recurrence: a multi-institutional analysis. Eur Urol.

[8] Ost P (2018). Surveillance or metastasis-directed therapy for Oligometastatic prostate Cancer recurrence: a prospective, randomized, multicenter phase II trial. J Clin Oncol.

[9] Palma DA, Olson RA, Harrow S, Gaede S, Louie AV, Haasbeek C (2018). Stereotactic Ablative Radiation Therapy for the Comprehensive Treatment of Oligometastatic Tumors (SABR-COMET): Results of a Randomized Trial. Int J Radiat Oncol Biol Phys.

[10] Yuan Y, Kishan AU, Nickols NG. Treatment of the primary tumor in metastatic prostate cancer. World J Urol. 2018. 10.1007/s00345-018-2552-8. 10.1007/s00345-018-2552-8 30456709

[11] Boeve LMS, et al. Effect on survival of androgen deprivation therapy alone compared to androgen deprivation therapy combined with concurrent radiation therapy to the prostate in patients with primary bone metastatic prostate Cancer in a prospective randomised clinical trial: data from the HORRAD trial. Eur Urol. 2019;75(3):410-418. 10.1016/j.eururo.2018.09.008. 10.1016/j.eururo.2018.09.008 30266309

[12] Parker CC, et al. Radiotherapy to the primary tumour for newly diagnosed, metastatic prostate cancer (STAMPEDE): a randomised controlled phase 3 trial. Lancet. 2018. 10.1016/S0140-6736(18)32486-3. 10.1016/S0140-6736(18)32486-3 PMC6269599 30355464

[13] O'Shaughnessy MJ (2017). A pilot study of a multimodal treatment paradigm to accelerate drug evaluations in early-stage metastatic prostate Cancer. Urology.

[14] Hu JC (2017). Increase in prostate Cancer distant metastases at diagnosis in the United States. JAMA Oncol.

[15] Calais J (2018). Impact of (68)Ga-PSMA-11 PET/CT on the Management of Prostate Cancer Patients with biochemical recurrence. J Nucl Med.

[16] Hope TA, et al. Meta-analysis of (68)Ga-PSMA-11 PET accuracy for the detection of prostate Cancer validated by histopathology. J Nucl Med. 2018. 10.2967/jnumed.118.219501. 10.2967/jnumed.118.219501 PMC6581235 30530831

[17] Calais J, Cao M, Nickols NG (2018). The utility of PET/CT in the planning of external radiation therapy for prostate Cancer. J Nucl Med.

[18] Calais J (2018). Potential impact of (68)Ga-PSMA-11 PET/CT on the planning of definitive radiation therapy for prostate Cancer. J Nucl Med.

[19] Oncology NRG, I. National Cancer (2020). Stereotactic Body Radiation Therapy in Treating Patients With Metastatic Breast Cancer, Non-small Cell Lung Cancer, or Prostate Cancer.

